# Strengthening Response Toward Promoting Mental Health in India: A Narrative Review

**DOI:** 10.7759/cureus.30435

**Published:** 2022-10-18

**Authors:** Abhilasha Dhyani, Abhay Gaidhane, Sonali G Choudhari, Sarvesh Dave, Swecha Choudhary

**Affiliations:** 1 Department of Dentistry and Public Health, School of Epidemiology and Public Health, Jawaharlal Nehru Medical College, Datta Meghe Institute of Medical Sciences, Wardha, IND; 2 Department of Community Medicine, School of Epidemiology and Public Health, Jawaharlal Nehru Medical College, Datta Meghe Institute of Medical Sciences, Wardha, IND; 3 Department of Oral Pathology, Triveni Institute of Dental Sciences, Hospital & Research Centre, Bilaspur, IND; 4 Department of Public Health, School of Epidemiology and Public Health, Jawaharlal Nehru Medical College, Datta Meghe Institute of Medical Sciences, Wardha, IND

**Keywords:** primary health care, universal health coverage, mental health illness, health promotion, mental health program

## Abstract

Mental health is an essential component of human development. It deals with human ideas and emotions, and it helps to lead a good life by paving the way for healthy minds. The absence of a healthy mind is a substantial hindrance to personal, societal, and national economic, political, and social functioning. For a long time in India, mental illness has been shrouded in stigma, ignorance, and superstition. The National Mental Health Program (NMHP) has undergone major strategic revisions throughout its existence, from instituting a district as the entity for program planning and implementation under the District Mental Health Program (DMHP) to integrating it with the National Rural Health Mission to productively scale up the program. Many researchers reviewed the program, which was also evaluated by governing and non-governmental institutions. Financial and human capital restrictions, a lack of public involvement, inefficient training, poor non-governmental organization/private cooperation, and a deficit of solid monitoring and evaluation system have all hampered the program's impact.

A thorough study of the literature on India's unique mental health initiatives was conducted using particular Medical Subject Heading (MeSH) terms, including “community mental health program,” “mental health project,” “innovative in mental health programs,” and “India,” and Boolean operators “AND/OR.” The MeSH keywords used were as follows: mental health project OR (“mental health project” [Mesh] OR “innovative in mental health programs” [Mesh]) AND community mental health program AND (“community mental health program” [Mesh]), India OR (“India” [Mesh]).

A preliminary search was conducted in Google Scholar and the PubMed database. A total of 55 indexed papers were found, of which 24 articles were duplicates, hence they were removed and the research eventually contained 31 investigations.

Over time, it has become clear that a strong focus on community mental health is critical, and that the DMHP and NMHP, in terms of coverage and utilization of their service components, need to be strengthened. As with many other public health programs, public awareness and information, education, and communication programs must be the most important components for change to occur at the community level. Many tactics and innovations also help to democratize mental health care by allowing the integration of mental health programs into primary care, which is more equitable in the long run and leads to “good mental health for everyone.”

## Introduction and background

The worldwide toll of mental, neurological, and drug-related illnesses (MNS) in terms of morbidity and premature mortality has been enormous [1]. According to a community-based epidemiological study conducted by the World Health Organization (WHO), prevalence rates of mental disorders in people range from 12.2% to 48.6% across their lifetimes and 8.4% to 29.1% over 12 months. MNS diseases are also responsible for 14% of the worldwide illness burden, as defined by disability-adjusted life years (DALYs). Despite the tremendous burden of MNS, WHO research found a substantial disparity between the incidence of mental disorders and the availability of care, with the global average of mental health practitioners being just nine per 100,000 people. Furthermore, the distribution of these items differs substantially between countries; low-income nations have less than one per 100,000 people, whereas high-income countries have more than 50 people. According to the WHO's Mental Health Atlas, per capita investment of lower and middle-income countries (LMICs) in mental health is also restricted [1]. Mental illnesses not only cause major suffering but also add to the nation's financial burden. Availability of mental well-being services is limited; a scarcity of professionals in psychological health, a lack of mental health perception, stigma, low education, and poverty, combined with the reluctance or incapacity of families to care for mentally sick individuals appear to be the most significant additional elements to the mental health burden. Furthermore, religious beliefs commonly associated with mental illness provide significant hurdles to seeking effective mental health care. The government has made several policies and programmatic steps to address these concerns, which have generated some improvements [2].

According to a large number of epidemiological surveys on mental conditions undertaken in the country, the mental burden of disease is predominant in both rural and urban areas of India [3]. Several non-governmental organizations (NGOs) have also initiated programs in the fields of rehabilitation, human rights in mental health, and school mental health. Despite these efforts and successes, much more has to be done in India to improve mental health in all aspects of society. Mental healthcare education, research, and diagnostic centers are required [3]. In psychiatry, significant scientific advancements have been made. Today, the majority of mental and behavioral illnesses may be successfully treated, and some can even be prevented. Most of these prevention, treatment, and cures are cost-effective. Even still, about two-thirds of those who have a recognized mental illness never seek medical attention [4,5].

## Review

Methodology

The literature search was conducted in PubMed, Scopus, EBSCO, and Google Scholar to search published articles in the English language using Medical Subject Heading (MeSH) terms, including “community mental health program,” “mental health project,” “innovative in mental health programs,” and “India”, and Boolean operators “AND/OR.” The MeSH keywords used were: mental health project OR (“mental health project” [Mesh] OR “innovative in mental health programs” [Mesh]) AND community mental health program AND (“community mental health program” [Mesh]), India OR (“India” [Mesh]). The studies that were included in the evaluation were those that looked at community-based mental health care or innovative mental healthcare initiatives in India. In total 55 indexed papers were found, of which 24 articles were duplicates, hence they were removed and the research eventually contained 31 investigations.

Global burden of mental health disorders

Mental and addiction problems impact a large fraction of the worldwide population, especially in high- and upper-middle-income countries, with a high burden. The frequency of mental disorders has increased in recent decades as a result of stigma and improper treatment. Mental and addiction problems affected about one billion people globally in 2016 [6]. They were responsible for 7% of all global disease burden measured in DALYs and 19% of all years spent incapacitated. Depression was linked to the majority of DALYs in both sexes, with greater rates in women than all other internalizing or emotional illnesses, and higher rates in men than other disorders such as drug use. It has been shown that four out of 10 people in the world population will suffer from a mental disease at some point in their lives. In 2010, mental and substance use conditions contributed to 183.9 million DALYs globally, accounting for 74% of all DALYs. Using published data, Vigo et al. [6] assessed the disease burden for mental illness, which revealed a larger estimate than previous estimates. According to the WHO's Global Burden of Diseases Report 2004, the number of DALYs lost owing to unipolar depressive disorder was 26.5 million (3.2%) in low-income countries and 29 million (5.1%) in middle-income countries. The same analysis predicted that by 2030, unipolar depressive illness would be the disease with the greatest DALY loss (6.2%). We should build healthcare services such that these therapies may be employed effectively in real-life situations. One way to do this is to think of mental diseases as long-term, frequently recurring problems, and then construct disease management techniques around that [7,8]. Suicide is the second leading cause of death in young people aged 15-29 years for both sexes, after road injury. More deaths were due to suicide in this age group than to interpersonal violence. For females and males, respectively, suicide is the second and third leading cause of death in this age group [9].

The burden of mental illness in India

In India, mental disorders are one of the primary etiologies of the non-fatal disease burden. In 2017, mental illnesses were the second most common cause of years lived with disability (YLDs) and the sixth most common cause of DALYs, posing a serious problem for healthcare systems, especially in developing countries. Mental health is becoming more generally acknowledged as a priority in global health policy, and it is now included in the United Nations' Sustainable Development Goals (SDGs). Recognizing the relevance of mental illnesses in decreasing the total disease burden, the United Nations has adopted the SDGs. To guarantee fair, inexpensive, and universal mental health treatment, India adopted its first National Mental Health Policy in 2014 and a revised Mental Healthcare Act in 2017. In India's federal structure, the states are largely crucial for health. Because of the social-economic and demographic diversity across India's states, tactics and treatments for reducing the prevalence of mental disorders must be adjusted to the specific needs of each state. In 2017, India had 197.3 million (95% of the total population) persons with mental disorders, accounting for 14.3% of the country's total population. In 2017, mental diseases accounted for 4.7% (3.7%-5.6%) of total DALYs in India, compared to 2.5% (2.0%-3.1%) in 1990. In 2017, 24 YLDs accounted for all DALYs from mental diseases, except for eating disorders, where YLDs accounted for 99.8% of DALYs. Mental illnesses were the major cause of YLDs in India in 2017, accounting for 14.5% of all YLDs. Depressive disorders (33.8%, 29.5-38.5) and anxiety disorders (19.0%, 15.9-22.4) contributed the most DALYs in India in 2017, followed by dissociative identity disorder (DID) (10.8%, 6.3%-15.9%), schizophrenia (9.8%, 7.7%-12.4%), bipolar disorder (6.9%, 4.9%-9.6%), and conduct disorder (5.9%, 4.9%-8.1%) [8].

Females contributed substantially more total DALYs than men due to depressive and disordered eating behaviors, whereas males contributed significantly more due to autism spectrum disorders and attention deficit hyperactivity disorders. Depressive disorders and anxiety disorders each had a crude prevalence of 3.3% (3.1%-3.6% for depressive disorders and 3.0%-3.5% for anxiety disorders), whereas bipolar disorders had a prevalence of 06% (0.5-0.7) and schizophrenia had a prevalence of 0.3% (0.2-0.3), which is one of the most common mental illnesses that strike people in their adult years. In India, 45.7 million of the population (42.4-49.8) suffered from depression in 2017. In the high socio-demographic index (SDI) state group, Tamil Nadu, Kerala, Goa, and Telangana had the highest prevalence of depressive disorders, followed by Andhra Pradesh in the intermediate SDI state group, and Odisha in the low SDI state group [8]. An estimated 57 million Indians (18% of the projected worldwide depressive population) live in India. Depression is anticipated to become more prevalent in India over the next few years as a result of the country's significant changes (such as those brought on by migration, urbanization, and modernization), which are happening at the same time as a fast sociodemographic shift [10]. According to the National Mental Health Survey (NMHS) 2015-16, India's mental morbidity rate for those over the age of 18 is now estimated at 10.6%, excluding diseases related to tobacco use. The examined population had a lifetime prevalence of 13.7% [11].

Consequences of mental economic health

The additional costs of mental diseases are substantially larger than the direct costs, according to the World Economic Forum (WEF), i.e., the negative economic consequences of not treating mental illness outweigh the costs of therapy. It appears that mental health and socioeconomic advancement are mutually beneficial. Investing in mental health is thus a development investment. The need of focusing on mental health is critical because the majority of those affected are between the ages of 25 and 44, indicating that the community's productive workforce is at risk [12]. Societies think that medication, health center visits, and hospitalization represent the actual cost of disease; however, the weight of illness, and particularly mental disorders, extends much beyond these "direct" screening and therapeutic expenditures. In its 2011 research on the global economic burden of non-communicable illnesses, the WEF emphasized three different methodologies for estimating economic disease burden, which not only represents the "hidden costs" of conditions but also their influence on overall economic growth. The human capital method distinguishes between direct and indirect costs when calculating the economic consequences of mental diseases and disease in general. Pharmaceutical, doctor visits, counseling sessions, hospitalizations, and other "visible costs" related to diagnosis and treatment in the healthcare system are sometimes referred to as "direct costs." The "invisible expenses" linked with monetary losses owing to indirect expenses include death, disability, and care-seeking, as well as lost production owing to employee absence or early retirement [13]. Public funds are well spent when timely and successful answers are provided to those who are experiencing mental health difficulties. Giving parents of young children high-quality parenting support, expanding access to psychological therapy, recognizing workplace distress early, diverting criminals with mental health concerns from jail, as well as assisting individuals with severe mental health issues in finding paid employment, all considerably enhance people's lives while generating both short- and long-term savings for government and the greater economy [14].

Existing mental health infrastructure in India

Both the government and other organizations have begun to provide community mental health services, notably since the National Mental Health Program (NMHP) was established in 1981. The major aim of the NMHP is to deliver basic psychological health care at the grassroots level, as well as to ensure that services are available and accessible to the most vulnerable and underprivileged people. Mental health abilities are being disseminated to the perimeter of the healthcare system, geographical resource allocation, and mental well-being collaboration treatment with general health services are some of the specific techniques. The National Institute of Mental Health and Neurosciences (NIMHANS) in Bangalore has started pilot research integrating mental health and community development through district mental health training programs in Bellary, Karnataka. In Goa, West Bengal, and Rajasthan, similar efforts have been undertaken [15]. In India, government spending on mental health accounts for only 0.06% of the total health expenditure, which accounts for barely 4% of the national gross national product (GNP). In India, there are 0.329 mental health outpatient services per 100,000 people. In general hospitals, there are 0.82 beds per 100,000 people. Only 43 mental hospitals with 1.469/100,000 beds and 0.047/100,000 psychologists and 0.301/100,000 psychiatrists exist in India. Qualified personnel is scarce; the availability of mental health nurses is 0.166/100,000, and that of social workers is 0.033/100,000 [14]. In India, mental health infrastructure is mostly restricted to huge, semi-permanent facilities that serve a small number of people [16]. The NMHS is projected to have a positive impact on mental health services across the country. Over 15% of Indian adults need active treatment for one or more mental health conditions, while mental health issues for both teenagers and the elderly are a major concern; metropolitan cities are seeing an increasing burden of mental health issues, especially among middle-aged working populations. In the long run, the consequences for employment, family life, and social interactions will be dire [17].

India's mental health-related initiatives

National Mental Health Program

In many ways, the Government of India's operation of the NMHP in August 1982 was a watershed moment in the country's psychiatric history. In 2012, we celebrated the 30th anniversary of this historic event [7]. In addition, the amended National Mental Health Policy of India and the draft National Health Policy of 2015 were announced in 2014 [13]. As a result, India became one of the world's earliest emerging nations to enact an NMHP [15].

The NMHP had the objective to ensure that everyone (particularly the most vulnerable and impoverished) has access to basic mental health treatment, to promote the use of mental health data in primary care and community welfare, and encourage community participation in the construction of mental health centers and community self-help programs. The District Mental Health Program (DMHP) provides community mental health services by combining mental health treatment at the primary care level with monitoring and help from a mental health unit at the district level, thus NMHP can achieve its goals. The paucity of mental health experts was due to the integration and delivery of mental health care through primary health care. Budgetary constraints and a lack of government financial backing contributed to the program's failure [18].

The program was primarily focused on treatments, with little emphasis on prevention and promotion. Suicide prevention, workplace stress management, and teenage counseling services, all of which could aid in community engagement and program efficacy, were also inadequate. Rather than primary prevention, a disease-focused strategy was considered [12]. The increased awareness and services offered to a bigger population are both beneficial characteristics of the NMHP. However, it should be recognized that the programs and efforts have not yet reached the general public. The importance of mental health in primary care has been recognized across the world, and concentrating efforts in this area has become a top priority [18,19].

We are still in the early stages of completely allowing patients, families, and communities to fulfill mental health's three goals of promotion, prevention, and treatment. These are the potential issues and solutions for the future [20].

Existing Government Policy and Programs on Mental Health in India

NMHP (1982), DMHP, manpower development schemes, modernization of state-run hospitals, upgradation of psychiatric wings of medical colleges/general hospitals, information education communication (IEC), training and research, and monitoring and evaluation are some of the existing government policies and programs on mental health.

National Mental Health Care Act (2013) includes the government's need to assure the right to access mental health care by all and it will be funded by the government. The government is required to fulfill manpower requirements according to international standards within 10 years, with the assurance of multiple rights of persons with illness, registration of health facilities as mental health establishments (hospitals with facilities for mental health care), banning of unmodified electro convulsion therapy (ECT), need of approval from Mental Health Review Board for ECT to minors, and exemption of general hospital psychiatry unit from the scope of this bill.

National Mental Health Policy (2014) includes the promotion of mental health, prevention of mental disorders and suicide, universal access to mental health services, enhanced availability of human resources for mental health, community participation, research, effective governance and accountability, monitoring, and evaluation [21].

Twelfth Five-Year Plan (2012-2017) and Mental Health

The Ministry of Health and Family Welfare (MOHFW) created a Mental Health Policy Group in 2012 to draft a DMHP for the 12th Five-Year Plan (2012-2017). The panel also summarized many of the outcomes of previous program reviews and created a draft for the DMHP with the goal to improve mental illness-related health and social consequences. The objectives were as follows: the primary goal for the 12th plan period is to reduce mental illness-related distress, disability, and premature mortality, as well as to improve rehabilitation from a mental condition, by assuring that psychiatric care is available and accessible to all, specifically the most marginalized and poor members of society. Other objectives were as follows: reduce stigmas, encourage community engagement, increase accessibility to preventative care for at-risk groups, safeguard persons with mental illness (PWMI) rights, and integrate mental health services with other programs such as rural and child health, motivate and empower employees, build administration, regulations, and accountability procedures to strengthen mental health service delivery infrastructure, develop awareness and information, and develop leadership, organizational, and accountability mechanisms [22]. These goals are now being pursued through extending community services and improving community-based programs (satellite clinics, school counseling, workplace stress management, and suicide prevention), organizing community awareness camps with the assistance of local groups, increasing national involvement (through collaboration with conscience and caretaker organizations), forming public-private partnerships with designated financial cooperation, establishing a special 24-hour hotline number (to notify the public about urgent mental health services, for example), assisting national and state mental health agencies in obtaining public funding, and so on. IEC efforts have been taken up in a standardized framework for the ongoing assessment of program activities and to monitor and execute the initiative, a centralized mental health team has been formed [23].

Program leadership is one of the most significant factors that have to be reconsidered to provide improved continuity and management at the local, state, and national levels. More dedication and coordination between the ministry of health, primary healthcare programs, and mental health experts would be required. Given the increased interest in primary rehabilitation services in India and throughout the world, previous policy and program issues, which are frequently comparable to those in other LMICs, should play a bigger role in shaping current policy [16].

National Mental Health Policy (2014)

The National Mental Health Policy lays out a prioritized plan to implement basic mental health care to all parts of the population across the country by 2020, within a realistic timeframe. The refocused NMHP, which was first created in 1982 and has five primary thrust areas, will be the tactical vehicle for implementing the strategy [24]. The DMHP has been modified to focus on a nodal institution, which in most cases is the zonal medical college [25]. The goals of the NMHP are as follows: improving secondary-level mental treatment facilities and strengthening medical colleges to generate psychiatric personnel; promoting the formation of general hospital psychiatry; substantial psychiatric institutions with a high percentage of long-stay patients needed optimizing and modernizing mental centers to turn them from primarily correctional settings to quality tertiary care centers with an active cultural focus for giving leadership in community mental health research and development; strengthening central and state mental health authorities to oversee ongoing facilitation, inter-sectorial coordination, and links with other national programs; prioritize tasks at the local/state level, and promote mental health programs, education, and research focusing on compiling a large database of epidemiological data on mental disorders and their progression/outcomes, community therapeutic needs, improved and more cost-effective stakeholder model, encouragement of trans-disciplinary study, and offering the conceptual foundation for well-being and policymaking. Focused IEC efforts, designed with the active participation of specialized organizations such as the National Institute of Mass Communication and focused on boosting community understanding, will be a critical component of this policy goal [14]. Similarly, the National Mental Health Policy aims to provide "the greatest good to the greatest number" by implementing five interconnected and mutually synergistic initiatives over the next two decades [18].

SDGs and Mental Health

In September 2015, the United Nations (UN) adopted mental health as one of the SDGs by recognizing the worldwide burden of mental disease and declaring that for the next 15 years, the mental state will be a top concern for international development, hence the UN made history [26]. The UN places mental health on the same footing as physical health in the initial declaration phase of the new SDGs and mandates that member nations achieve fairness and universal access to health care. Psychological health and well-being advancement, universal health coverage, and availability of high healthcare coverage are all worthy goals. Mental health is mentioned three times in goal 3 (the "health goal"), including in the target "through prevention and treatment, reduce the fatality rate from non-communicable illnesses by one-third & improve mental state and well-being."

The UN has adopted WHO recommendations and made a firm statement for mental well-being in global progress in the overarching goals of this development agenda. This is a watershed moment for psychological well-being, which has been long campaigned for by global mental health experts and non-governmental organizations like fundamentals SDG [25]. In the new SDGs, the UN has ultimately identified mental stability as a preference for globalization, paving the way for an enormous approach to address the nation's problems over the next 15 years [26]. All 17 SDGs are intended to be met by 2030 when nations are required to organize their efforts to fulfill their goals. Several SDG objectives and targets are directly or indirectly tied to health. Goal 3 includes the following objectives: a one-third reduction in non-communicable disease mortality should be achieved by 2030 through the promotion of mental health and well-being, and the prevention and treatment of substance misuse, including the use of alcohol and narcotics. This includes a focus on substance abuse prevention and treatment programs and all forms of violence against women and girls, including sexual and other forms of exploitation, must be eliminated in both the public and private spheres [27,28].

Mental health promotion and protection

When people live in environments that support mental health and encourage healthy lives, they are more likely to succeed in their goals. An environment that values and defends fundamental civil, political, economic, and cultural rights is necessary for mental health promotion. It is important that national mental health policies not just focus on mental diseases, but also acknowledge and address the larger concerns that promote mental wellness. Mental health promotion should be integrated into policies and programs in both the public and private sectors. Promoting mental health necessitates cross-sector collaboration, interventions in the early years of life, improved access to education and microcredit programs for women's economic empowerment, and laws and efforts against discrimination, rights, opportunities, and care for those with mental problems should be promoted [29]. Primary care providers need to be knowledgeable of the socioeconomic determinants of poor health, such as debt, unemployment, housing issues, marital issues, and abuse of alcohol, cigarettes, and drugs. Once recognized, issues can either be handled directly when practical, or typically by directing them to other pertinent organizations and partnering with possibilities for community development [30,31].

## Conclusions

NMHP has been working for the past three decades and several lessons from different projects and programs have been learned and insights have been derived to determine the plan of action. Planned leadership at all levels, may it be governing or administering the projects or arranging financial and human resources, has always been an important determinant in the success of the program. Community participation, IEC activities, participation of NGOs in all sectors, proper training of the health professionals, and a sustainable method and implementation mechanism also play a role in the success and outcome of a program. NHMP on one hand has many features similar to most health programs but has certain peculiarities as well. The most common top-to-bottom approach for health planning has sadly proved to be ineffectual, and a paradigm shift toward the bottom-to-up approach is seen to be effective. Modern medical care for mental health appears to be absent in the community and the major reason behind it is the acceptance of local quacks and quackeries in the community, as they have been proving remedies related to mind and spirit for ages in addition to other prevalent factors like ignorance and the superstition of the locals. They have been prevalent in the community for ages and poor literacy levels and lack of adequate scientific education lead to their acceptance at a broad level. In rural places, public understanding and attitude toward mental illness may not imply a desire for contemporary medical assistance. Innovations in approach involving technology will only make it more accessible and easy for the community members and will empower them to provide care for others in the community. The various strategies and innovations also promote the democratization of mental health care and allow the integration of mental health programs as primary care, which in long term is equitable and achieves a perspective that is "good mental health for all."
